# A Comprehensive Review on the Effects of Vegetarian Diets on Coronary Heart Disease

**DOI:** 10.7759/cureus.29843

**Published:** 2022-10-02

**Authors:** Funmilola Babalola, Ayobami Adesuyi, Favour David, Benedicta-B A Kolajo, Alexsandra Urhi, Omotola Akinade, Adewale M Adedoyin, Gabriel Alugba, Abimbola E Arisoyin, Obiamaka P Okereke, Ojali R Unedu, Adeyinka O Aladejare, Aduwa A Oboasekhi, Gibson O Anugwom

**Affiliations:** 1 Epidemiology and Public Health, Texas Department of State Health Services, San Antonio, USA; 2 Community Medicine, Babcock University Teaching Hospital, Ilishan-Remo, NGA; 3 Medicine, College of Medicine, University of Ibadan, Ibadan, NGA; 4 Clinical Sciences, Windsor University School of Medicine, Cayon, KNA; 5 Psychiatry, Federal Neuro-Psychiatric Hospital, Benin City, NGA; 6 Internal Medicine, General Hospital Ikorodu, Ikorodu, NGA; 7 Internal Medicine, Lagos State University Teaching Hospital, Ikeja, NGA; 8 Internal Medicine, Delta State University, Abraka, NGA; 9 Internal Medicine, College of Medicine, University of Lagos, Lagos, NGA; 10 Family Medicine, Ebonyi State University, Abakaliki, NGA; 11 Internal Medicine, University of Jos, Jos, NGA; 12 Epidemiology, University of Texas Health Science Center at Houston, Houston, USA; 13 General Practice, Evercare Hospital Lekki, Lekki, NGA; 14 Menninger Department of Psychiatry and Behavioral Sciences, Baylor College of Medicine, Houston, USA

**Keywords:** vegetarianism, plant-based diet, cardio vascular disease, vegetarian diet, coronary heart disease (chd)

## Abstract

Coronary heart disease (CHD) is one of the leading causes of morbidity and mortality worldwide. Dietary modifications in the form of a vegetarian diet can perhaps be the key to the prevention and management of cardiovascular diseases. The aims of this review are to determine the association between a vegetarian diet and CHD, to compare the risk of CHD in different types of vegetarian diets, and to assess variability in the biochemical predictors of CHD in the various vegetarian diets. Our study inferred that adherence to a plant-based diet was inversely related to the incidence of heart failure risk. Our research further supports the idea that a vegetarian diet is advantageous for the secondary prevention of CHD since it alters lipid profiles, lowers body mass index (BMI), and increases plasma antioxidant micronutrient concentrations. Additionally, eating a plant-based diet starting in adolescence is linked to a decreased risk of cerebrovascular disease (CVD) by middle age. An increase in sensitization and education efforts is imperative to ensure that people are appropriately informed about this option to significantly improve their quality of life.

## Introduction and background

Coronary heart disease (CHD) is the leading cause of morbidity and mortality in the United States (US) and worldwide. According to estimates, 85.6 million Americans have cardiovascular disease (CVD), and the number is continuing to rise [1]. Healthy lifestyle choices may reduce the risk of myocardial infarction by more than 80% with nutrition playing a key role [2].

The refusal to eat meat (red meat, poultry, seafood, and the flesh of any other animal) is known as vegetarianism [3]. Vegetarians may be classified as vegans, pesco-vegetarians, lacto-vegetarians, lacto-ovo-vegetarians, and flexitarians [4,5]. Vegans avoid using or eating any animal products [4,5]. Pesco-vegetarians consume fish and other seafood [4,5]. Lacto-vegetarians eat dairy products; lacto-ovo-vegetarians eat dairy products and eggs [4,5]. Flexitarians occasionally or even once a week eat meat [4,5]. A plant-based diet is low in cholesterol, fat, animal products, salt, and sugar [6]. By way of dietary advice, well-planned vegetarian diets should be promoted as having advantages for preventing and reversing atherosclerosis and lowering risk factors for CVD [2]. 

Growing research points to health benefits and possible cardiovascular advantages of plant-based diets and eating habits that prioritize plant-based foods while reducing animal products [7]. Many studies have discovered that plant-based diets, particularly those abundant in high-quality plant foods including whole grains, fruits, vegetables, and nuts, are linked to a decreased risk of cardiovascular events and intermediate-risk factors [7,8]. The objective of this review is to determine the association between a vegetarian diet and CHD.

Over time, a lot of studies have been carried out on the prevalence of CHDs and various factors that predispose people of different races and ages to these diseases. Various modifications have been implicated over time in reducing the incidence and prevalence of these diseases. One of these is the application of a vegetarian diet.

## Review

Methodology

Search Strategy

This review article was conducted using the scale for the assessment of non-systematic review articles (SANRA). We searched two databases: EMBASE (Excerpta Medica database) and PubMed (MEDLINE) using specific search terms. Search terms used were “vegetarian diet” AND “ischemic heart disease” AND “cardiovascular disease”. We searched for recent articles; hence, we used articles written from 2012 to 2022.

Inclusion Criteria

Original articles in the English language, from 2012 to 2022, related to the study's objective were included.

Exclusion Criteria

Review and commentary articles, articles older than 10 years, and articles not written in English language were excluded.

Results

**Figure 1:**
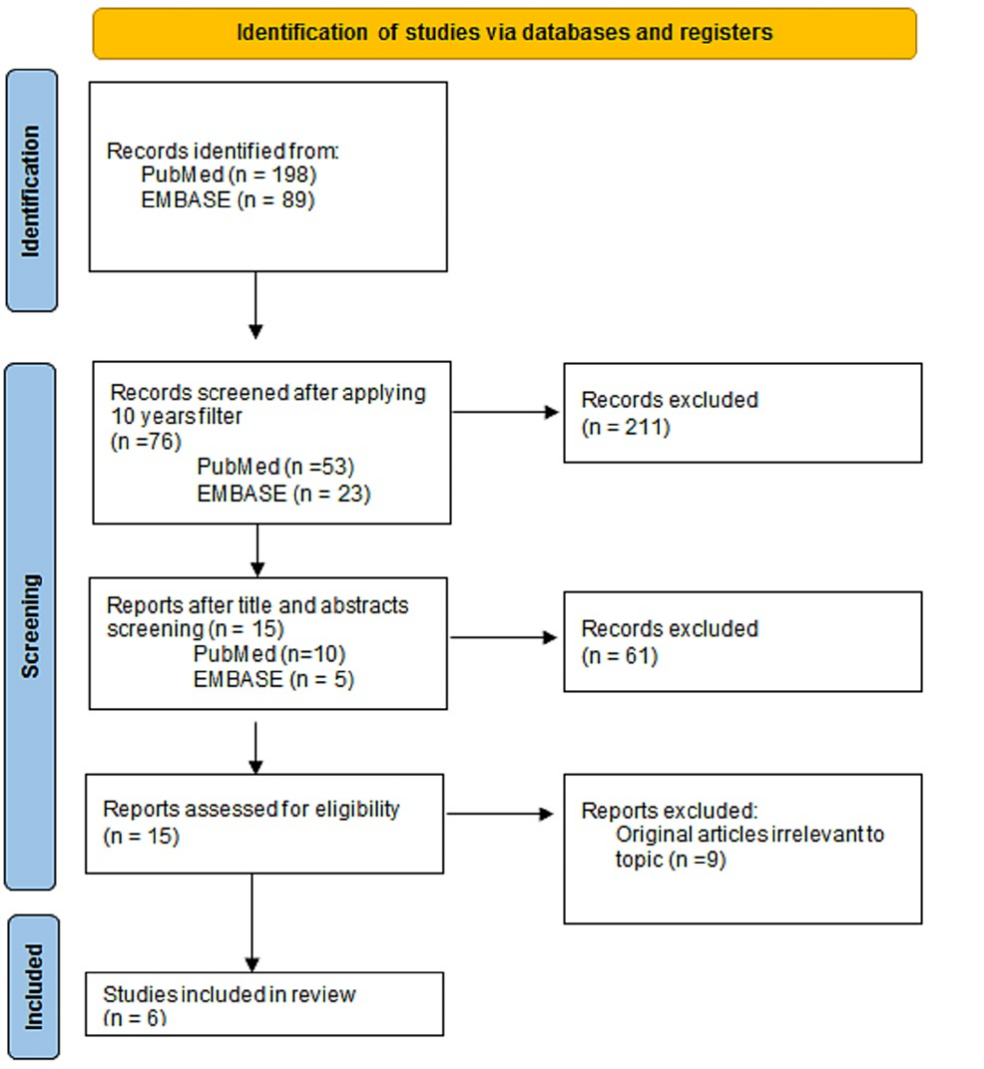
Flow diagram showing the selection process of included articles used in this review

Our data search returned a total of 287 articles. These were screened for relevance to the objective, which resulted in six articles (Figure 1). Four of the six articles were observational studies and the other two were randomized studies. The articles reviewed provided the effects of vegetarian diet on CHD. The articles also revealed that adherence to a plant-based diet was inversely related to the incidence of heart failure risk and that vegetarian diet is beneficial for secondary prevention of CAD via modulation of lipid profile, reduction in BMI, and patients having a high concentration of plasma antioxidants micronutrients in their system. Also, it was seen that consumption of a plant-centered diet starting in young adulthood is associated with a lower risk of CVD by middle age (Table 1).

**Table 1:** The characteristics and summary of findings of articles included in this review CAD; coronary artery disease; AHA: American Heart Association; hs-CRP: highly-sensitive C-reactive protein; PCI; percutaneous coronary intervention; LDL-C: low-density lipoprotein cholesterol; CARDIA: Coronary Artery Risk Development in Young Adults; MPO: myeloperoxidase; MMP: metalloproteinase

Author/Year/Title	Study Design	Study Population /Sample Size	Diet and targeted outcome	Summary	Limitation of study
1. Shah et al, 2018 [1].	A randomized, open-label, blinded end point study design.	100 participants from New York University Langone Medical Center with a history of angiographically defined CAD underwent 1:1 randomization to either a vegan diet or the AHA-recommended diet.	Vegan diet versus the AHA-recommended diet. Outcome: hs-CRP concentration, inflammatory markers, white blood cell cellular adhesion molecules, anthropometric data, glycemic markers, lipid profiles, and quality of life as measured by the EuroQol 5 dimensions questionnaire	It showed a considerably higher decrease in hsCRP with a vegan diet compared to the AHA's diet recommendation. However, there was no significant difference in the degree of weight loss and waist circumference	The study was not powered to assess for differences in major adverse cardiovascular and cerebrovascular events, also participants may have underreported their intake on the food records.
2. Djekic D et al, 2020 [9].	A randomized, open-label, cross-over study	Participants with CAD treated with PCI and on optimal medical therapy/ 27 participants	A lacto-ovo-vegetarian diet allowing the intake of eggs and dairy products. Outcome: oxidized LDL-C selected cardiometabolic risk factors, gut microbiota, and plasma metabolome.	This study revealed that a vegetarian diet compared with a diet including daily meat consumption improved plasma lipid profile, particularly triacylglycerol, phosphatidylcholine, alkylphosphatidylcholine, and sphingomyelin in coronary artery disease. Results of this study support that a vegetarian diet may be beneficial for secondary prevention of CAD via modulation of lipid profile.	Small study size and the few women subjects in the study which may decrease generalizability.
3. Wright N et al, 2017 [10].	A prospective, two-arm, parallel, superiority study	Ages 35–70, from a general practice in Gisborne, New Zealand, diagnosed with obesity or overweight and at least one of type 2 diabetes, ischaemic heart disease, hypertension, or hypercholesterolemia/ 65 participants	Low-fat plant-based diet. Outcome: BMI and dyslipidemia	The programme led to significant improvements in BMI, cholesterol, and other risk factors. It also achieved greater weight loss at 6 and 12 months than any other trial that does not limit energy intake or mandate regular exercise.	The study population had a higher number of females and a higher mean age.
4. Choi Y et al, 2021 [11].	Prospective study	Participants were 4946 adults in the CARDIA prospective study.	Plant-centered diet. Outcome: incident Cerebrovascular disease	Consumption of a Plant-centered diet, starting in young adulthood is associated with a lower risk of CVD by middle age.	The nature of the observational study design, unmeasured or residual confounding could not be ruled out.
5. Navarro et al, 2016 [12].	Observational cross-sectional study	329 Male volunteer	Vegetarian diet (lacto-ovo-vegetarian, lacto-vegetarian or vegan) versus Omnivores/ matrix metalloproteinases-2 and 9	The study found significantly lower concentrations of MPO, MMP-9, MMP-2, and MMP-9/TIMP-1 ratio in VD compared to omnivores (all P-value < 0.05).	Causal inference may be limited due to its cross-sectional study design.
6. Lara KM et al, 2019 [13].	Prospective cohort study	16,068 participants (mean age 64.0 + 9.1 years)	1. Convenience dietary pattern (more on meat dishes, pasta, Mexican dishes, pizza, fried potatoes, Chinese dishes, and fast food). 2. Plant-based pattern (more on cruciferous vegeta- bles and other vegetables, fruit, beans, and fish). 3. Sweets/fats pattern (more on desserts, bread, sweet breakfast foods, chocolate, candy, solid fats and oils, and miscella- neous sugar). 4. Southern pattern, (more on fried food, organ meats, processed meats, eggs, added fats, and sugar’sweetened beverages). 5. Alcohol/salads (more on wine, liquor, beer, leafy greens and salad dressing). Outcome: Incident heart failure	Adherence to a plant-based dietary pattern was inversely associated with incident HF risk, whereas the Southern dietary pattern was positively associated with incident HF risk.	Misclassification from inaccuracies of reporting dietary intake in the food frequency questionnaire (FFQ) likely occurred. The potential for residual confounding and a study population that did not include individuals with race/ethnicity other than non-Hispanic black or white may have altered and/or limited the generalizability of the results.

Positive effects of a vegetarian diet on the lipid profile of CHD patients

In a randomized cross-over study by Djekic et al., it was discovered that subjects with ischemic heart disease (IHD) experienced a reduction in oxidized low-density lipoprotein cholesterol (LDL-C) after being placed on a vegetarian diet for four weeks [9]. There was also a reduction in their cardiometabolic risk factors compared to their counterparts on an isocaloric meat diet (meat diet of the same calorie) [9]. This reduction in oxidized LDL-C has been attributed to the presence of a particular baseline gut microbiota rich in several genera of the families Ruminococcaceae and Barnesiellaceae in these individuals [9]. These gut microbes play important roles in the clearance of intestinal infections and immunomodulation [14]. Ordinarily, the conversion of LDL-C to its oxidized form enhances the formation of fatty streaks and the formation of atherosclerotic plaques [15]. People who suffer from IHD have a reasonably high level of oxidized LDL-C than people free from IHD [14]. Thus, even when on medical therapy, a vegetarian diet help lowers the level of oxidized LDL-C in people with IHD. This was confirmed when four weeks of a vegetarian diet lowered the level of oxidized LDL-C in subjects with IHD with a meat diet, who were also being treated with percutaneous coronary intervention (PCI) [9]. Furthermore, coronary artery disease (CAD) patients on standard medical therapy, who were placed on a four-week vegetarian diet showed a favorable and significant impact on plasma lipids, particularly sphingomyelins (SMs), alkyl phosphatidylcholine (O-PC), phosphatidylcholine (PC), and triglycerides (TGs) compared to isocaloric meat diet. Additionally, data from high-throughput lipidomics connected a vegetarian diet to the presence of long-chain polyunsaturated TGs in high concentrations and the absence of lipotoxic lipids such TGs with saturated fatty acyl chains [16]. According to another study, CAD patients had lower amounts of unsaturated TGs in their epicardial adipose tissue than persons without the condition [17]. Generally, vegetarian diet improves plasma lipid profile by reducing the level of lipotoxic lipids species.

The positive effect of long-term plant-centered diet consumption on the incidence of CVDs

In another prospective cohort study conducted by Choi et al., a plant-centered over the long term was linked to a 52% decreased risk of incident CVD in people who were tracked since young adulthood [11]. Additionally, a 13-year rise in the quality of a plant-based diet was linked to a 61% decreased risk of CVD occurrences in the next 12-year period [11]. However, since there are other risk factors relevant to the incidence of CVDs, the timing and length of exposure to these risk factors may differ in how this illness manifests in adults. As a result, an assessment in middle or advanced age may not provide a comprehensive view of the whole spectrum of illness development in adulthood. This study demonstrated a link between a higher quality plant-based diet starting in early adulthood and a decreased risk of CVDs in adulthood [11]. Social parameters like race and educational background were also found to be mediators of the relationship between a plant-based diet and CVD incidence. A proposed mechanism of how a plant-based diet may reduce CVDs incidents is the trapping of free radicals which leads to a reduction in reactive oxygen molecules thereby preventing tissue damage. This successful endeavor has been linked to substances like phenolics, carotenoids, tocopherols, and ascorbic acid, which are plentiful in nuts and seeds, fruits, vegetables, and whole grains [18].

Association between whole food plant-based diet and reduction in BMI, cholesterol, and other risk factors for CADs

In a randomized controlled study using low-fat food plant-based diet in a community for obesity, IHD, or diabetes done by Wright et al., a reduction in BMI, cholesterol, and other risk factors was achieved [10]. The dietary approach included whole grains, legumes, vegetables, and fruits [10]. Participants were advised to eat until satiation and no restriction on total energy intake was placed. Participants were asked to not count calories. A diet chart was provided to participants outlining which foods to consume, limit, or avoid. Starches such as potatoes, sweet potato, bread, cereals, and pasta were also encouraged to satisfy their appetite and they were asked to avoid refined oils (e.g. olive or coconut oil), animal products (meat, fish, eggs, and dairy product, high-fat plant foods such as nuts and avocados, and highly processed foods. Participants were encouraged to minimize sugar, salt, and caffeinated beverages [10]. Daily vitamin B12 (methylcobalamin) supplements (50 μg) were also provided for participants. This study was said to have had better weight reduction in six and 12 months compared to studies that do not impose calorie restrictions and frequent activity requirements. Participants in this study were focused on a whole food plant-based diet and this was attributed to the low energy density in the food consumed [10].

Comparative studies on the effects of a vegan diet and the American Heart Association-recommended diet on high-sensitivity C-reactive protein, markers of inflammation, and glucometabolic markers in patients with CHD

Shah et al, contrasted the effects of the American Heart Association's (AHA) recommended diet on CHD with those of a vegan diet in a prospective study design [1]. In patients with established CHD receiving medical treatment that followed guidelines, this research showed a considerably higher decrease in highly-sensitive C-reactive protein (hs-CRP) with a vegan diet compared to the AHA's diet recommendation. A risk indicator for serious negative cardiovascular outcomes in CHD is hs-CRP [19]. However, there was no significant difference in the degree of weight loss and waist circumference [1]. In a study of 46 patients with CHD who were assigned to a one-month vegan diet regimen with prepared meals and stress management, it was established that there was a resulting decrease in plasma cholesterol [20]. A study analyzed the outcomes of the MultiSite Cardiac Lifestyle Intervention Program [19] and encompassed 56 CHD patients and 75 patients at risk for CHDs using a low-fat, plant-based diet, exercise, whole foods, stress management, and group support sessions. Over the course of the three months of this intervention, it was seen that waist-hip ratio, CRPs, BMI, insulin concentration, and lipid profile all decreased.

Effects of vegetarian diet on circulating biomarkers of CVD in apparently healthy vegetarian men

Navarro et al. demonstrated that a vegetarian diet is associated with decreased concentration of myeloperoxidase (MPO), metalloproteinase (MMP-9 and MMP-2), and tissue inhibitor of MMP (TIMP-1)/MMP-9 ratio when compared with omnivores in apparently healthy individuals [12]. The reduced concentration of these cardiovascular biomarkers has been linked to a high intake of fruits and vegetables with a reduced concentration of circulating neutrophils and leucocytes in vegetarians compared to omnivores. In metabolic syndrome and diabetes, there is an associated high concentration of leucocytes, which is also associated with high activity of MMP, cardiovascular dysfunction, and remodeling [12]. This study reiterates the association between a high intake of vegetarian meals and its associated reduced biomarkers of CVDs.

Bioactive compounds and their effects on lipid profile

Cengiz1 et al., were able to elaborate on the general fact that a vegetarian diet reduces the risk of CVDs, a fact related to low saturated fat and cholesterol content [21]. Soy protein contains isoflavones and polyphenols, which are bioactive compounds that have been implicated in the reduction of low-density lipoprotein (LDL) levels which is important in atherosclerosis pathogenesis [22]. Studies on Isoflavones have shown that this compound is responsible for arterial vasodilation and the reduction of serum cholesterol in animal models [23]. It also inhibits atherosclerosis in postmenopausal monkeys [23]. It has been shown that vegetarian diets lower blood pressure and deaths from CHD and stroke decline when blood pressure levels drop [21]. 

Improved endothelial function and vegetarian diet consumption

According to Kahleova et al., the advantages of a vegetarian diet include lowering CVD risk factors and benefits in preventing atherosclerosis [2]. Blood vessels are lined with the endothelium, which helps in regulating angiogenesis and vascular tone as well as preventing leucocyte adhesion. Various adverse factors have been implicated in abnormal endothelial function; some of these are sedentary lifestyle, western diet type, hypertension, and inflammation. In a nutshell, a diet rich in meat has been associated with compromised endothelial function while high fruit and vegetable intake is associated with improved endothelial function [24]. As a matter of fact, the compromised endothelial function has been noted to improve with a vegetarian diet. Apart from CRP, other inflammatory biomarkers like interleukin-6 and soluble intercellular adhesion molecule-1 have been shown to reduce in the serum with plant based-diet [25]. These inflammatory biomarkers have been implicated in various CVDs, thus, plant based-diet plays a positive role in reversing the pathophysiology of these diseases. Increased level of trimethylamine N-oxide (TMAO) has also been associated with the risk of myocardial infarction, stroke, or even death [26]. An organic substance produced by the gut bacteria is called TMAO and is a culprit which promotes atherosclerosis through the accumulation of cholesterol in foam cells [26]. Dietary phosphatidylcholine and carnitine, which are plentiful in a variety of food sources, such as eggs, dairy products, and red meat, are then used in its hepatic metabolism of it. Vegetarians' gut microbiome generates less triethylamine, which is the precursor of TMAO, thus, a consequential reduction in the incidence of CVDs [27]. 

Strengths and limitations

This review demonstrated its strength in its ability to explore the effects of a vegetarian diet on CHD. Across all selected articles, the impact of the reduction in the risk factors associated with CHD was also demonstrated. The limitations observed include the following: studies conducted in clinical settings could have observer bias because of the possible influence of the researcher's expectations. Also, the causal relationship between a vegetarian diet and CHD could not be appreciated in the included articles that were observational studies. Another limitation was the attrition effect, as most of the patients were lost to follow-up and they may be underreporting dietary intake among participants. There is a need to use a population size that reflects the effects of a vegetarian diet on CHD across race, sex, and socioeconomic classification.

Recommendations

An increase in sensitization and education efforts is imperative to ensure that people are appropriately informed about this great option to improve their quality of life significantly. Beyond education, however, is the issue of accessibility. Good quality, organic whole foods are very expensive and most times outside the budget range of most families, and these disparities are even more glaring when you examine them by racial demographics. Working on subsidizing the prices of good quality foods to improve accessibility in addition to education will go a long way towards encouraging more people to adopt a vegetarian or vegan diet.

## Conclusions

In a world where the incidence and prevalence of atherosclerosis and CVD are on the increase with all the subsequent health challenges, poor quality of life, and dependence on polypharmacy just to get through the day, it is perhaps refreshing to see that dietary modifications in the form of a vegetarian diet can perhaps be the key to prevention and management of cardiovascular diseases. The advantages of a whole food plant-based diet can never be overemphasized. It has been studied extensively in this work and has been found to be of great benefit to improving outcomes in people with CVD and reducing the markers in people at risk of developing it. A slow steady progression to a generalized plant-based lifestyle might just be the key to reducing the incidence of CVD and improving outcomes for those already afflicted. The logistics of how to make this happen would need to be studied extensively so that it can be applied in a timely fashion within the next few decades.
